# Methyl 6-chloro­nicotinate

**DOI:** 10.1107/S1600536811053517

**Published:** 2011-12-17

**Authors:** Yong Xu, Ling-Ling Yang, Sheng-Yong Yang, Jie Liu

**Affiliations:** a State Key Laboratory of Biotherapy and Cancer Center, West China Hospital, West China Medical School, Sichuan University, Chengdu 610041, People’s Republic of China; b Department of Pharmaceutical and Bioengineering, School of Chemical Engineering, Sichuan University, Chengdu 610065, People’s Republic of China

## Abstract

The mol­ecule of the title compound, C_7_H_6_ClNO_2_, is almost planar, with a dihedral angle of 3.34 (14)° between the COOMe group and the aromatic ring. In the crystal, the mol­ecules are arranged into (1
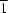
2) layers by C—H⋯N hydrogen bonds and there are π–π stacking inter­actions between the aromatic rings in adjacent layers [centroid–centroid distance 3.8721 (4) Å]

## Related literature

For background to the synthesis of methyl 6-chloro­nicotinate, see: González *et al.* (2009 ▶); Rekha *et al.* (2009 ▶). For a related structure, see: Ma & Liu (2008 ▶).
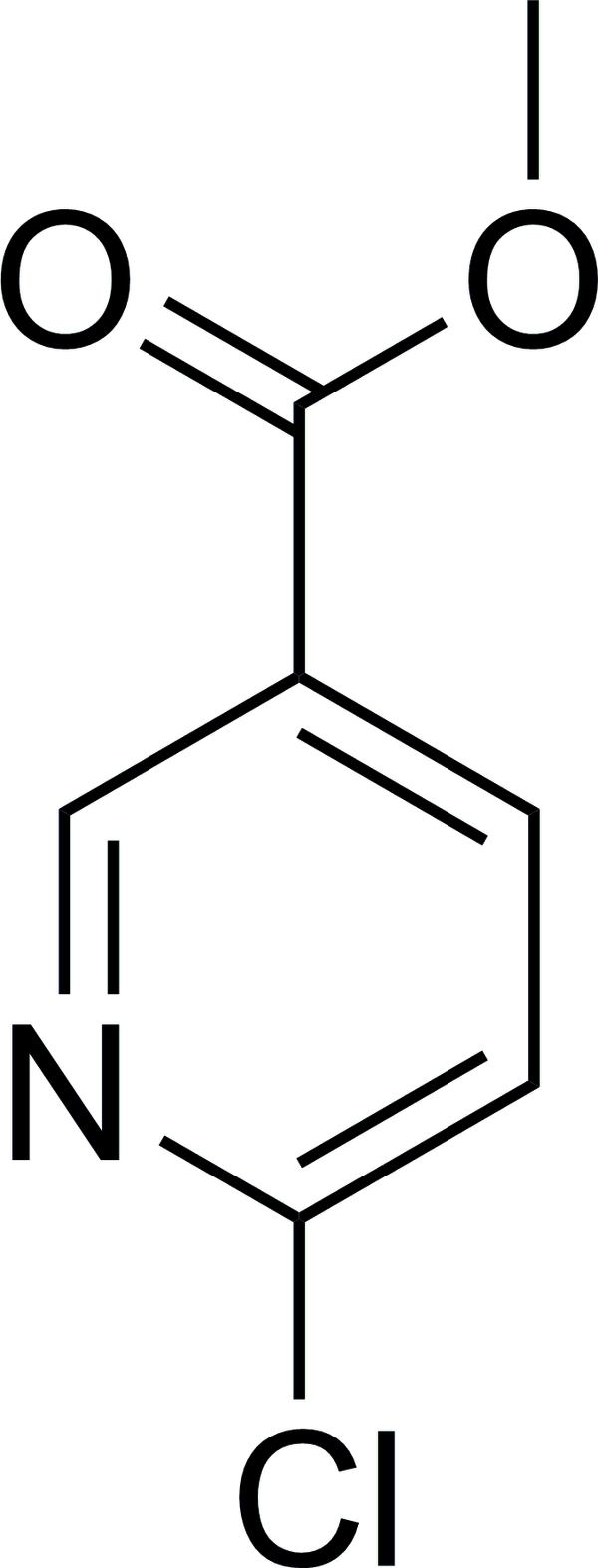


## Experimental

### 

#### Crystal data

C_7_H_6_ClNO_2_
*M* *_r_* = 171.58
Triclinic, 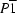

*a* = 3.8721 (4) Å
*b* = 5.8068 (6) Å
*c* = 17.3721 (18) Å
α = 95.563 (9)°
β = 94.918 (8)°
γ = 104.657 (9)°
*V* = 373.64 (7) Å^3^
*Z* = 2
Mo *K*α radiation
μ = 0.45 mm^−1^
*T* = 293 K
0.30 × 0.30 × 0.12 mm

#### Data collection

Oxford Diffraction Xcalibur E diffractometer
Absorption correction: multi-scan (*CrysAlis PRO*; Agilent, 2011 ▶) *T*_min_ = 0.037, *T*_max_ = 1.000
3068 measured reflections
1527 independent reflections
855 reflections with *I* > 2σ(*I*)
*R*_int_ = 0.029

#### Refinement

*R*[*F* ^2^ > 2σ(*F* ^2^)] = 0.055
*wR*(*F* ^2^) = 0.119
*S* = 0.99
1527 reflections
101 parameters
H-atom parameters constrained
Δρ_max_ = 0.23 e Å^−3^
Δρ_min_ = −0.18 e Å^−3^

### 

Data collection: *CrysAlis PRO* (Agilent, 2011 ▶); cell refinement: *CrysAlis PRO*; data reduction: *CrysAlis PRO*; program(s) used to solve structure: *SHELXS97* (Sheldrick, 2008 ▶); program(s) used to refine structure: *SHELXL97* (Sheldrick, 2008 ▶); molecular graphics: *OLEX2* (Dolomanov *et al.*, 2009 ▶) and *Mercury* (Macrae *et al.*, 2006 ▶); software used to prepare material for publication: *OLEX2*.

## Supplementary Material

Crystal structure: contains datablock(s) global, I. DOI: 10.1107/S1600536811053517/gk2439sup1.cif

Structure factors: contains datablock(s) I. DOI: 10.1107/S1600536811053517/gk2439Isup2.hkl

Supplementary material file. DOI: 10.1107/S1600536811053517/gk2439Isup3.cml

Additional supplementary materials:  crystallographic information; 3D view; checkCIF report

## Figures and Tables

**Table 1:** Hydrogen-bond geometry (Å, °)

*D*—H⋯*A*	*D*—H	H⋯*A*	*D*⋯*A*	*D*—H⋯*A*
C3—H3⋯N1^i^	0.93	2.59	3.440 (4)	151

Symmetry code: (i) 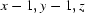.

**Fig. 1.**
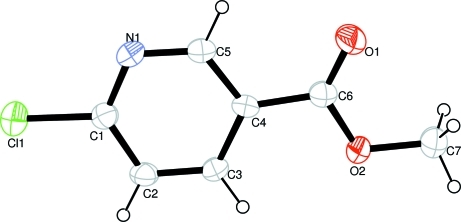
The molecular structure of the title compound with displacement ellipsoids drawn at the 30% probability level.

**Fig. 2.**
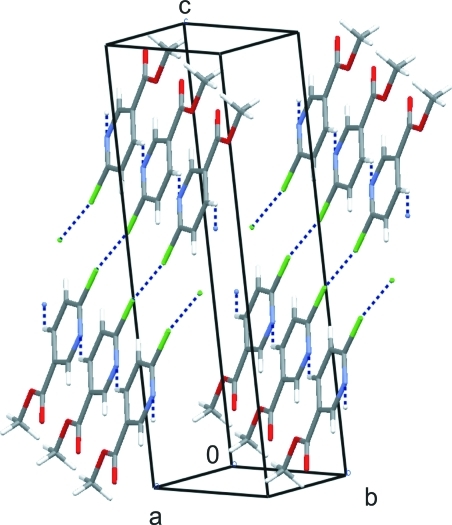
A packing diagram of the title compound. Intermolecular interactions are shown as dashed lines in blue.

## supplementary crystallographic information

### Comment

The title compound is one of the key intermediates in our synthetic investigations of GPCR(G-protein coupled receptor) modulators. We have synthesized the title compound and here we report its crystal structure.

As shown in Fig.1, the molecule is nearly planar, the dihedral angle formed by the pyridine ring and the ester group (C6/C7/O1/O2) being 3.34 (14)°. Weak C—H···O and C—H···N hydrogen bonds are present in the crystal structure linking molecules into (1 -1 2) layers. There are also π-π stacking interactions between the aromatic rings in adjacent layers [centroid-centroid distance 3.8721 (4) Å].

### Experimental

The title compound was prepared by the following method. A mixture of 6-chloronicotinic acid (5.67 g, 0.036 mol), dimethyl carbonate (10.95 mL, 0.131 mol) and concentrated H_2_SO_4_ (2.72 mL, 0.049 mol) was refluxed for 17 h. Then aqueous NaHCO_3_ solution (8.6 g in 86 mL water) was added, extracted with dichloromethane (150 mL), dried (Na_2_SO_4_), filtered and evaporated under reduced pressure to afford the title compound.  Crystals suitable for X-ray analysis were obtained by slow evaporation from dichloromethane solution at room temperature over a period of one week.

### Refinement

H atoms were positioned geometrically and refined using a riding model approximation, with d(C—H) = 0.93 - 0.96 Å, and *U*_iso_(H) =1.2*U*_eq_(C) or 1.5*U*_eq_(methyl C).

### Crystal data

**Table:**

C_7_H_6_ClNO_2_	*Z* = 2
*M**_r_* = 171.58	*F*(000) = 176
Triclinic, *P*1	*D*_x_ = 1.525 Mg m^−^^3^
*a* = 3.8721 (4) Å	Mo *K*α radiation, λ = 0.7107 Å
*b* = 5.8068 (6) Å	Cell parameters from 741 reflections
*c* = 17.3721 (18) Å	θ = 3.6–26.3°
α = 95.563 (9)°	µ = 0.45 mm^−^^1^
β = 94.918 (8)°	*T* = 293 K
γ = 104.657 (9)°	Block, colourless
*V* = 373.64 (7) Å^3^	0.30 × 0.30 × 0.12 mm

### Data collection

**Table:**

Oxford Diffraction Xcalibur E diffractometer	1527 independent reflections
Radiation source: Enhance (Mo) X-ray Source	855 reflections with *I* > 2σ(*I*)
graphite	*R*_int_ = 0.029
Detector resolution: 16.0874 pixels mm^-1^	θ_max_ = 26.4°, θ_min_ = 3.6°
ω scans	*h* = −4→4
Absorption correction: multi-scan (*CrysAlis PRO*; Agilent, 2011)	*k* = −7→7
*T*_min_ = 0.037, *T*_max_ = 1.000	*l* = −21→21
3068 measured reflections	

### Refinement

**Table:**

Refinement on *F*^2^	Primary atom site location: structure-invariant direct methods
Least-squares matrix: full	Secondary atom site location: difference Fourier map
*R*[*F*^2^ > 2σ(*F*^2^)] = 0.055	Hydrogen site location: inferred from neighbouring sites
*wR*(*F*^2^) = 0.119	H-atom parameters constrained
*S* = 0.99	*w* = 1/[σ^2^(*F*_o_^2^) + (0.041*P*)^2^] where *P* = (*F*_o_^2^ + 2*F*_c_^2^)/3
1527 reflections	(Δ/σ)_max_ < 0.001
101 parameters	Δρ_max_ = 0.23 e Å^−^^3^
0 restraints	Δρ_min_ = −0.18 e Å^−^^3^

### Special details

**Table:**

Geometry. All e.s.d.'s (except the e.s.d. in the dihedral angle between two l.s. planes) are estimated using the full covariance matrix. The cell e.s.d.'s are taken into account individually in the estimation of e.s.d.'s in distances, angles and torsion angles; correlations between e.s.d.'s in cell parameters are only used when they are defined by crystal symmetry. An approximate (isotropic) treatment of cell e.s.d.'s is used for estimating e.s.d.'s involving l.s. planes.
Refinement. Refinement of *F*^2^ against ALL reflections. The weighted *R*-factor *wR* and goodness of fit *S* are based on *F*^2^, conventional *R*-factors *R* are based on *F*, with *F* set to zero for negative *F*^2^. The threshold expression of *F*^2^ > σ(*F*^2^) is used only for calculating *R*-factors(gt) *etc*. and is not relevant to the choice of reflections for refinement. *R*-factors based on *F*^2^ are statistically about twice as large as those based on *F*, and *R*- factors based on ALL data will be even larger.

### Fractional atomic coordinates and isotropic or equivalent isotropic displacement parameters (Å^2^)

**Table:**

	*x*	*y*	*z*	*U*_iso_*/*U*_eq_	
Cl1	0.3480 (2)	1.23478 (16)	0.44450 (4)	0.0710 (4)	
O1	0.4875 (6)	0.7116 (4)	0.10003 (12)	0.0728 (8)	
O2	0.1351 (5)	0.3994 (4)	0.14496 (10)	0.0521 (6)	
N1	0.5039 (6)	1.1467 (5)	0.30475 (15)	0.0529 (7)	
C1	0.3350 (8)	1.0448 (6)	0.36075 (16)	0.0452 (8)	
C2	0.1561 (7)	0.8050 (6)	0.35567 (17)	0.0480 (8)	
H2	0.0435	0.7421	0.3971	0.058*	
C3	0.1498 (7)	0.6630 (6)	0.28773 (15)	0.0443 (8)	
H3	0.0331	0.5003	0.2823	0.053*	
C4	0.3182 (7)	0.7630 (5)	0.22726 (15)	0.0399 (7)	
C5	0.4935 (7)	1.0035 (5)	0.23934 (17)	0.0484 (8)	
H5	0.6125	1.0704	0.1993	0.058*	
C6	0.3266 (8)	0.6273 (6)	0.15097 (18)	0.0464 (8)	
C7	0.1289 (8)	0.2550 (6)	0.07211 (16)	0.0618 (10)	
H7A	−0.0050	0.3080	0.0315	0.093*	
H7B	0.0171	0.0899	0.0765	0.093*	
H7C	0.3703	0.2710	0.0599	0.093*	

### Atomic displacement parameters (Å^2^)

**Table:**

	*U*^11^	*U*^22^	*U*^33^	*U*^12^	*U*^13^	*U*^23^
Cl1	0.0898 (7)	0.0583 (7)	0.0610 (6)	0.0171 (5)	0.0109 (5)	−0.0070 (5)
O1	0.0875 (17)	0.0632 (18)	0.0575 (14)	−0.0044 (13)	0.0288 (13)	0.0043 (13)
O2	0.0669 (14)	0.0379 (14)	0.0472 (12)	0.0064 (11)	0.0127 (10)	−0.0011 (10)
N1	0.0611 (17)	0.0362 (17)	0.0564 (16)	0.0033 (13)	0.0081 (13)	0.0050 (14)
C1	0.0451 (18)	0.043 (2)	0.0472 (17)	0.0109 (16)	0.0029 (14)	0.0064 (16)
C2	0.0517 (19)	0.044 (2)	0.0505 (18)	0.0091 (16)	0.0165 (15)	0.0146 (16)
C3	0.0457 (17)	0.0346 (19)	0.0485 (17)	0.0021 (14)	0.0071 (14)	0.0060 (15)
C4	0.0394 (17)	0.042 (2)	0.0400 (16)	0.0105 (15)	0.0059 (13)	0.0138 (14)
C5	0.0509 (19)	0.042 (2)	0.0496 (17)	0.0048 (16)	0.0110 (14)	0.0112 (16)
C6	0.0450 (18)	0.046 (2)	0.0489 (18)	0.0112 (16)	0.0075 (15)	0.0093 (17)
C7	0.071 (2)	0.054 (2)	0.0551 (19)	0.0092 (18)	0.0116 (17)	−0.0019 (18)

### Geometric parameters (Å, °)

**Table:**

Cl1—C1	1.728 (3)	C3—H3	0.9300
O1—C6	1.198 (4)	C3—C4	1.382 (4)
O2—C6	1.333 (4)	C4—C5	1.376 (4)
O2—C7	1.444 (3)	C4—C6	1.482 (4)
N1—C1	1.322 (4)	C5—H5	0.9300
N1—C5	1.333 (3)	C7—H7A	0.9600
C1—C2	1.380 (4)	C7—H7B	0.9600
C2—H2	0.9300	C7—H7C	0.9600
C2—C3	1.367 (4)		
			
C6—O2—C7	116.0 (2)	C5—C4—C6	118.1 (3)
C1—N1—C5	116.2 (3)	N1—C5—C4	124.2 (3)
N1—C1—Cl1	115.3 (2)	N1—C5—H5	117.9
N1—C1—C2	124.6 (3)	C4—C5—H5	117.9
C2—C1—Cl1	120.1 (2)	O1—C6—O2	123.3 (3)
C1—C2—H2	121.1	O1—C6—C4	124.1 (3)
C3—C2—C1	117.8 (3)	O2—C6—C4	112.6 (3)
C3—C2—H2	121.1	O2—C7—H7A	109.5
C2—C3—H3	120.2	O2—C7—H7B	109.5
C2—C3—C4	119.5 (3)	O2—C7—H7C	109.5
C4—C3—H3	120.2	H7A—C7—H7B	109.5
C3—C4—C6	124.3 (3)	H7A—C7—H7C	109.5
C5—C4—C3	117.7 (3)	H7B—C7—H7C	109.5

### Hydrogen-bond geometry (Å, °)

**Table:**

*D*—H···*A*	*D*—H	H···*A*	*D*···*A*	*D*—H···*A*
C3—H3···N1^i^	0.93	2.59	3.440 (4)	151
C5—H5···O1	0.93	2.49	2.812 (3)	101

Symmetry codes: (i) *x*−1, *y*−1, *z*.
